# Acne in Cystic Fibrosis: Tips for Prescribing Isotretinoin in the Genomodulatory Era

**DOI:** 10.1155/crdm/5545924

**Published:** 2026-05-12

**Authors:** Julia O’Mahony, David Mullane, Cathal O’Connor

**Affiliations:** ^1^ Dermatology, South Infirmary Victoria University Hospital, Cork, Ireland, sivuh.ie; ^2^ Paediatric Respiratory Medicine, Cork University Hospital, Cork, Ireland, hse.ie; ^3^ INFANT Research Centre, University College Cork, Cork, Ireland, ucc.ie

**Keywords:** acne, ADEK supplementation, cystic fibrosis, isotretinoin, ivacaftor, lumacaftor, retinoid, tezacaftor, vitamin A

## Abstract

A 16‐year‐old boy with cystic fibrosis (CF) on fat‐soluble vitamin (including vitamin A) supplementation develops severe acne. He is commenced on isotretinoin, a vitamin A derivative. The package insert says that vitamin A supplementation should be stopped, presenting a therapeutic dilemma. We review the literature and previous reports of isotretinoin use in patients with CF to provide guidance on how to successfully treat acne while minimizing adverse effects, including in patients on genomodulatory therapy.

## 1. Case Report

A 16‐year‐old boy with cystic fibrosis (CF) develops severe, scarring, nodulocystic acne (Investigator Global Assessment Scale 4) unresponsive to topical agents and oral tetracyclines. He is on elexacaftor/tezacaftor/ivacaftor for CF and vitamin ADEK and pancreatic enzyme supplementation for CF‐related pancreatic exocrine insufficiency. How can dermatologists effectively and safely prescribe isotretinoin in this situation? Isotretinoin was initiated at a low dose of 10 mg (0.15 mg/kg) and cautiously increased to a maximum of 60 mg (0.9 mg/kg) over 10 months, achieving a cumulative dose of 130 mg/kg. Complete clearance was seen, with the only adverse effect being cheilitis. Two‐monthly liver function and triglyceride levels were normal. Following advice from our pediatric gastroenterology colleagues and as recommended by the isotretinoin package insert, vitamin ADEK supplementation was held (and Vitamin DEK supplementation was continued). Vitamin A levels were checked every two months. After 6 months of therapy, vitamin A was low (1.0 μmol/L, normal range: 1.4–3.84 umol/L), and ADEK supplementation was reinstituted. The case prompted us to review the logistics of prescribing isotretinoin for CF in the genomodulatory era.

## 2. Discussion

CF is caused by pathogenic variants in the CF transmembrane conductance regulator (*CFTR*) gene [1], characterized by respiratory, pancreatic, and liver dysfunction, with malfunctioning chloride ion transport across epithelial cell membranes causing thickened mucus. Exocrine pancreatic insufficiency can impact fat digestion and absorption. Therefore, supplementation with fat‐soluble vitamins ADEK is prescribed for patients with CF, with monitoring of serum vitamin A levels. ^1^ Genomodulatory therapy with *CFTR* modulators has revolutionized outcomes in CF [1]. *CFTR* modulation can normalize fat‐soluble vitamin absorption, rendering ADEK supplementation unnecessary. However, acneiform eruptions have been reported with *CFTR* modulators due to altered sebum production, skin microbiome alteration, or hypersensitivity reactions [2–4].

Isotretinoin is a vitamin A derivative indicated for acne treatment [5]. It works by reducing sebum production, shrinking sebaceous glands, and inhibiting keratinocyte proliferation. In addition to malabsorption of fat‐soluble vitamins, CF‐related liver disease or abnormal liver function can impact isotretinoin metabolism and increase the risk of hepatotoxicity. There is also a higher than average risk of baseline mucocutaneous dryness, hypertriglyceridemia, and myalgia with CF [1]. Patients with CF commonly have lower body mass. Dosing of isotretinoin in patients with CF has been debated due to these CF‐specific issues [6]. Lower doses (0.25–0.5 mg/kg/day) for longer periods (9–12 months) may be effective and better tolerated [5]. Guidelines suggest that blood monitoring (liver function and lipid profile) should be checked prior to initiation of isotretinoin and once at peak dose, but reviews have suggested that blood monitoring is not required in healthy young patients without a personal or family history of liver disease or hypertriglyceridemia [7]. It has also been recommended that supplementation with vitamin A should be avoided in patients with CF while on isotretinoin to avoid vitamin A toxicity [6]. We suggest that, during isotretinoin therapy in patients with CF, vitamin A levels are monitored with liver function and triglycerides at baseline, then every 2–3 months during their course (Table 1). Fat‐soluble vitamin supplementation can be continued and reviewed on the basis of blood monitoring.

**TABLE 1:** Suggestions for prescribing isotretinoin in patients with cystic fibrosis (CF) based on review of the limited literature and expert opinion.

Point	Suggestion
Patients with CF can typically tolerate isotretinoin therapy with similar outcomes to patients without CF	Consider isotretinoin at all levels of acne severity in patients with CF
New genomodulatory therapies are life‐changing for patients with CF but have a common side effect of acneiform eruptions	Consider isotretinoin for acne in patients with CF on genomodulatory therapy
Patients with CF often have excessive exposure to antibiotics and colonization with antibiotic‐resistant organisms	Consider isotretinoin as an alternative to topical or oral antibiotics at all levels of acne severity in patients with CF
Patients with CF have impaired fat‐soluble (including A) vitamin absorption, although genomodulatory therapy may lead to normalization of vitamin A levels and eliminate the need for fat‐soluble vitamin supplementation	Monitor vitamin A levels at baseline and every 2–3 months in patients with CF on isotretinoin therapy. Amend fat‐soluble vitamin supplementation in a personalized manner in patients with CF based on vitamin A blood monitoring
Both CF and isotretinoin can be associated with liver dysfunction and pancreatic dysfunction	Monitor liver function and lipid profile at baseline and every 2–3 months in patients with CF on isotretinoin therapy. Counsel all patients and parents on the rare side effects of liver dysfunction and pancreatitis secondary to hypertriglyceridemia and educate them on signs of these complications
Patients with CF may have baseline mucocutaneous dryness or other symptoms, which may be exacerbated by isotretinoin therapy	Start at a more conservative dose (e.g. 0.25 mg/kg/day) and escalate dosing more slowly in patients with CF. Counsel all patients on common side effects of isotretinoin.

Antibiotic therapy can be considered as an alternative to isotretinoin in patients with CF with acne. However, longer‐term courses of oral antibiotics such as tetracyclines and macrolides used for treating acne can be detrimental in CF due to antibiotic resistance, which may limit treatment options for infective exacerbations of CF‐related bronchiectasis [1].

**FIGURE 1:**
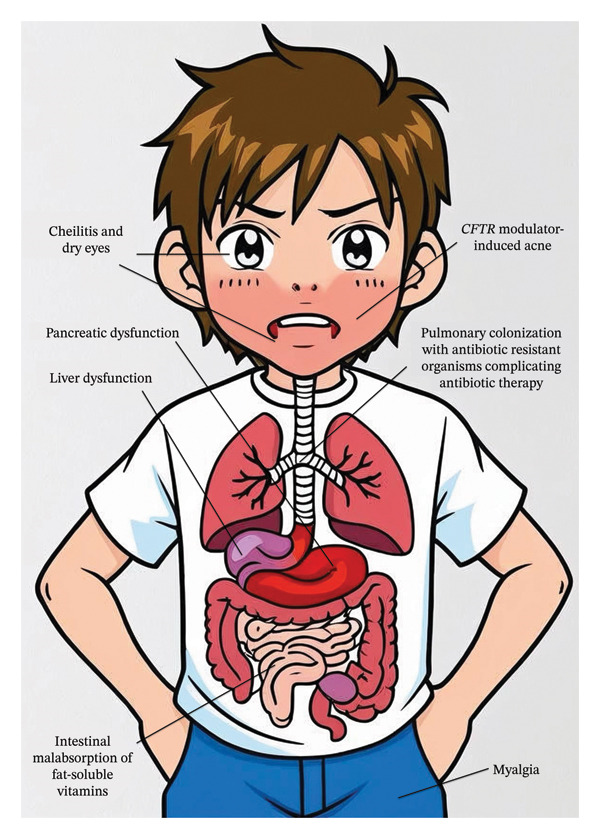
The underlying problems associated with cystic fibrosis (CF), which may complicate the prescription of isotretinoin in patients who have CF and acne.

Isotretinoin therapy should not be contraindicated in patients with CF, but enhanced monitoring of vitamin A levels, liver function, and lipid profiles should be undertaken under specialist dermatology care. We provide guidance to clinicians for managing patients with CF who require isotretinoin (see Figure 1).

## Author Contributions

Julia O’Mahony, Cathal O’Connor, and David Mullane managed the patient. Cathal O’Connor and David Mullane conceived of the manuscript idea. Julia O’Mahony wrote the first draft. Julia O’Mahony, Cathal O’Connor, and David Mullane reviewed and approved subsequent and final drafts.

## Funding

The authors have nothing to report.

## Ethics Statement

The patient and guardians of the patient in this manuscript have given written informed consent/assent for participation in the study and the use of their de‐identified, anonymized, aggregated data and their case details (including photographs) for publication.

## Consent

Please see the Ethics Statement.

## Conflicts of Interest

The authors declare no conflicts of interest.

## Data Availability

The data that support the findings of this study are available on request from the corresponding author. The data are not publicly available due to privacy or ethical restrictions.
